# Chronic Endodontic Infections and Cardiovascular Diseases: Does the Evidence Support an Independent Association?

**DOI:** 10.7759/cureus.19864

**Published:** 2021-11-24

**Authors:** Yaser A Aloutaibi, Abdulaziz S Alkarim, Esraa M Qumri, Lolo A Almansour, Faisal T Alghamdi

**Affiliations:** 1 Advanced General Dentistry, The University Dental Hospital, King Abdulaziz University, Jeddah, SAU; 2 Oral Biology, Faculty of Dentistry, King Abdulaziz University, Jeddah, SAU

**Keywords:** outcome assessment, systemic disease, chronic endodontic infection, coronary heart disease, endodontics, cardiovascular disease, periapical lesions, apical periodontitis

## Abstract

Previous studies have shown that endodontic infections might increase the risk of cardiovascular diseases. However, there is no conclusive evidence that endodontic infections are associated with cardiovascular diseases among individuals with cardiac conditions. This systematic review aimed to collect and evaluate the current evidence on the relationship between chronic endodontic infections and cardiovascular diseases.

The PubMed, Scopus, and Web of Science databases were searched, and Google Scholar was used to retrieve relevant clinical studies within the past 10 years (2011-2021). Observational studies (prospective cohort, retrospective cohort, cross-sectional, and case-control studies), which investigated the impact of endodontic infections and apical periodontitis in individuals with cardiac conditions at risk of cardiovascular disease, in English were considered. Review papers, duplicates, animal studies, and other irrelevant studies were excluded. Four investigators independently carried out the study selection and data collection processes. Quality assessment was performed in this review. Fourteen studies with 960,652 human subjects were included in this review. No association between endodontic infections and cardiovascular diseases among individuals with cardiac conditions was noted. Most of the studies showed a moderate overall risk of bias by 57.14% (n=8).

There is weak evidence regarding the association between cardiovascular diseases and chronic endodontic infections. Further longitudinal clinical studies are required to determine the association between cardiovascular diseases and endodontic infections.

## Introduction and background

One of the main causes of death worldwide is cardiovascular disease (CVD), accounting for 17.9 million (32%) fatalities worldwide in 2019 [1]. The association of periodontal disease with atherosclerosis and CVD is widely recognized, and data from observational studies support this association [2]. However, only a few studies have examined the correlation between pulpal infection and CVD and have yielded inconsistent findings [3].

Two important studies that investigated root canal therapy (RCT) as a surrogate for pulpal infection found that individuals who had undergone RCT had a greater risk of coronary heart disease than those who did not [4,5]. These studies were restricted in their assessment of RCTs based on self-reports, which might lead to exposure misclassification. It was also difficult to determine whether the increased CVD risk was caused by the treatment of pulpal inflammation or by the inflammatory condition itself [4,5].

There is evidence for many epidemiologic markers related to periodontitis and CVD. According to the most recent consensus report on periodontitis and CVD, there is preliminary evidence for an association between coronary heart disease and marginal periodontitis [6]. The factors underlying such a correlation indicate that oral bacterial species can infiltrate the circulatory system and promote bacteremia [7,8], and the presence of oral bacteria in atheromatic lesions has also been established [9].

Similarly, there is a widespread problem that alternative-origin oral cavity-related chronic inflammatory diseases could be a contributing factor to the pathogenesis of CVD. Infection of the root canals and apical tissues after pulp necrosis, which is referred to as "apical periodontitis," is among the most problematic clinical features documented. Apical periodontitis (AP) is an inflammatory condition induced by bacterial invasion inside the root canal system, which results in radiolucency due to apically framed bone deterioration and inflammatory periapical tissue response, which can be seen on periapical radiographs [10,11]. However, this could be associated with increased systemic concentrations of reactive peripheral blood cells or inflammatory mediators, thus affecting overall cardiovascular health [10,11].

A recent observational study has sought to explore particular associations between endodontic infections and CVD, although substantial associations are difficult to identify because of the obvious risk of bias and challenges regarding validity and reliability reported in those investigations [12]. The latest umbrella review has highlighted four systematic reviews on the subject that involved published studies until four years ago, with only one of those performing a meta-analysis of four investigations [13]. However, most significantly, the available systematic reviews have indeed been classified as having critical low-to-moderate quality in terms of methodological/reporting discrepancies used during the process of the review [13].

The current effort to assess and summarize different large epidemiological data in the subject is considered necessary and proper to offer an unmistakable description of endodontic infections and their correlation with CVD, pursuing extremely thorough and diaphanous methodology, while also providing additional insight. Furthermore, considering the wide range of causality effects and methodological restrictions of the involved studies, evidence regarding this association may serve as a valuable benchmark concerning future predicted risk variables to be considered extensively when calculating individual or integrated impacts on CVD. Thus, the objective of the current systematic review was to thoroughly gather and evaluate the current evidence on the association between CVD and chronic endodontic infections.

## Review

Methods

Study Protocol and Research Question

A systematic review of studies on the association between CVD and endodontic infections was conducted in accordance with the Preferred Reporting Items for Systematic Reviews and Meta-Analyses (PRISMA) guidelines [14]. The following research question for the current systematic review was considered: does the evidence support an independent association between endodontic infections and cardiovascular disease in young or adult cardiac patients in any type of observational study?

Literature Search Methodology

An electronic search was conducted in February 2021 and updated on June 28, 2021, on the following databases: PubMed (2011-2021), Scopus (2011-2021), Web of Science (2011-2021), and Google Scholar (2011-2021). The following search keywords were used: [(chronic apical periodontitis) OR (apical periodontitis) OR (tooth periapical lesion) OR (tooth apical lesion) OR (radiolucent periapical tooth lesion)] AND [(chronic tooth inflammation) OR (chronic pulp tooth inflammation) OR (endodontic infection) OR (tooth pulp infection) OR (chronic dental infection)] AND [(root canal tooth therapy) OR (endodontic treatment) OR (tooth root canal treatment)] AND [(coronary heart disease) OR (coronary disease) OR (atherosclerosis) OR (stroke) OR (myocardial infarction) OR (cardiovascular disease) OR (CVD) OR (hypertension)].

Selection Criteria

Several following inclusion criteria were established: (1) observational studies, including retrospective cohort, prospective cohort, case-control, and cross-sectional studies were conducted and involved human subjects; (2) research articles published in English; (3) research articles published between 2011 and 2021; (4) studies including any form of periapical or periradicular lesion suggesting endodontic infection and inflammation of apical tissues (sometimes described as "apical periodontitis"), as verified clinically/radiographically through CVD patient file records; and (5) studies involving any result related to CVD, prevalence, or incidence of the disorder, including, but not limited to, stroke, coronary heart disease, vascular/arterial disease, and myocardial infarction. Several following categories of publications were excluded: (1) review papers; (2) in vitro/in vivo studies, case reports, editorial, or opinion articles; (3) studies demonstrating the clinical significance of CVD in dental fields other than endodontics; and (4) studies investigating non-human sources.

Study Selection

After excluding duplicate publications, studies were chosen by screening the title and abstract, and full-text evaluation was performed separately by four reviewers. A senior reviewer settled any disagreements among the reviewers. To identify further suitable studies, the references provided in the retrieved full-text papers were also evaluated.

Data Extraction and Synthesis

Following the study selection method, five independent reviewers recognized and classified the available material in the selected studies. Data extraction was carried out using prepiloted standardized forms by reviewers who were not visible to the study's origin or author identity. Information inputs were specifically associated with study identification, sample size, study design, outcomes, exposure/condition, and additional study-specific comments.

The following information was presented in a single table: author, study design, country, year, patient age (mean and standard deviation), number of participants, source of infection and assessment tools, main outcomes, and any supplementary comments.

Quality Assessment

Five reviewers independently assessed the methodological risk of bias in each included study. The methodological quality of each study was performed using the risk of bias assessment tool outlined in the Cochrane risk of bias tool (Risk-of-bias VISualization (robvis)-ROBINS-I tool) to evaluate the internal validity of the included studies in light of the latter's expected observational design [15]. For each study's quality evaluation, the following parameters were considered: (1) confounders, (2) selection of participants, (3) classification of interventions, (4) deviations from intended interventions, (5) missing data, (6) measurement of outcomes, and (7) selection of the reported result. Using the robvis visualization tool, the risk of bias was assigned as “Critical” (brown dots), “Serious” (red dots), “Moderate” (yellow dots), “low” (green dots), and “no information” (blue dots). The total risk of bias was then assessed for each included paper, considering the following prior analyses: (1) low risk of bias (studies that fulfilled at least five or six of the seven quality criteria); (2) moderate risk of bias (studies that met just one or two of the quality criteria); and (3) serious risk of bias (studies that met only one or two of the quality criteria). Depending on how the other parameters were evaluated, the existence of at least one yellow dot or red dot resulted in a moderate or serious risk of bias, respectively.

Primary and Secondary Outcome Measurements

Studies discussed the association between chronic endodontic infection/apical periodontitis (AP) with cardiovascular diseases (CVDs) as primary outcomes. However, studies with clear evidence of endodontic/apical periodontitis lesions and informing on any hard cardiovascular outcomes (lesion of endodontic origin {LEO} and CVDs), excluding alternative measures, were considered secondary outcomes.

Statistical Analysis

No meta-analysis was performed owing to the heterogeneity among the included studies in this review. The results were synthesized descriptively as complementary data.

Results

Study Selection

**Figure 1:**
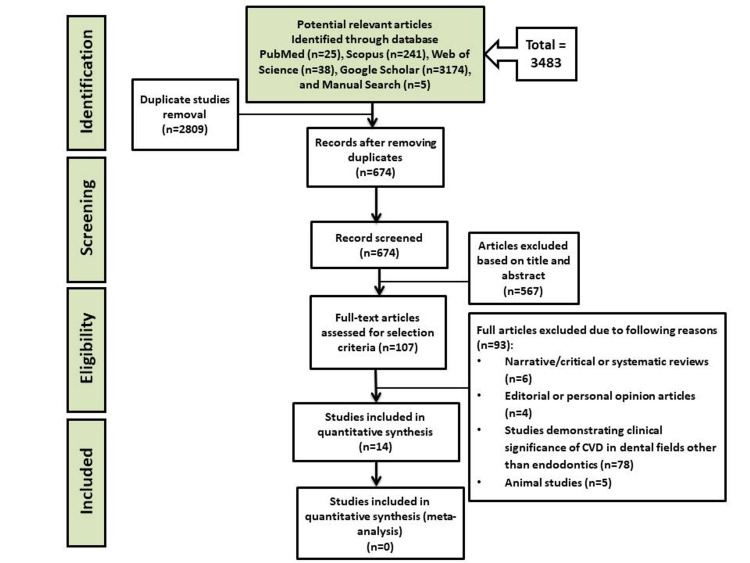
Preferred Reporting Items for Systematic Reviews and Meta-Analyses (PRISMA) flowchart for study selection CVD: cardiovascular disease

During the initial title and abstract evaluation, the article search returned 3483 entries from all databases. After eliminating duplicate data, the remaining 674 articles were screened. In total, 567 articles were eliminated on the basis of their abstract and title. After screening, 107 full-text articles were determined to be eligible. Owing to various reasons, only 93 papers were eliminated from our review. Only 14 studies were eventually involved in the systematic review. Figure 1 depicts a flow chart describing the search for articles in this systematic review.

Study Characteristics

Fourteen human studies chosen for the review were conducted within the past 10 years (2011-2021) and met all the inclusion criteria but none of the exclusion criteria [16-29]. These studies investigated the evidence for a relationship between endodontic infection/apical periodontitis and CVD. All studies included in this systematic review were observational studies and were distributed as follows: 10 were cross-sectional studies, two were retrospective cohort studies, one was a prospective cohort study, and one was a case-control study. The studies included in this systematic review were from the following different countries: Austria, Brazil, Finland, Germany, Italy, India, Taiwan, and the United States. The total sample in this review comprised 960,652 patients ranging from 55 to 666,768 patients. Regarding gender, one study included only male patients, whereas the rest included both male and female patients (Table 1) [17]. The reported age of the subjects in nine of the included investigations was greater than 45 years, with five studies reporting younger age ranges in combination with older or only young age ranges [17-20,25].

**Table 1:** Results of observational studies assessing the association between chronic endodontic infections and cardiovascular diseases CS: cohort study; CSS: cross-sectional study; M: male; F: female; AP: apical periodontitis; hsCRP: high sensitivity C-reactive protein; FMD: flow-mediated dilatation; CVD: cardiovascular disease; CAD: coronary artery disease; CHD: coronary heart disease; EP: endodontic pathology; c-IMT: intima-media thickness; ET: endodontic treatment; RCT: root canal therapy; HCA: hypercholesterolemia; LEO: lesions of endodontic origin; AMI: acute myocardial infarction; UA: unstable angina

Author	Country	Study design	Year	Number of participants	Patient age (mean±SD)	Source of infection and assessment tool	Main outcomes of cardiovascular disease	Additional comments
Cowan et al. [16]	United States	Prospective CS	2020	n=6274; 2966 M/3308 F	62.3±5.7 years for non-ET and 62.7±5.7 years for ET cases	Self-reported history of ET	Incidence of CHD	Diabetes, periodontal disease, ET as a proxy for infection, and self-reported diseases were also found in a portion of the sample.
Chauhan et al. [17]	India	CSS	2019	n=120; 120 M only	Only range between 20 and 40 years	AP, radiographic assessment	FMD, c-IMT	Early CVD physiologic and anatomic measurements.
Messing et al. [18]	United States	CSS	2019	n=666,768	Only range between 18 and 65 years	EP	Prevalence all CVDs combined (self-reported)	Study of epidemiologic and genetic associations
Garrido et al. [19]	Chile	CSS	2019	n=55; 32 M/23 F	Cases: 25.9±5.0 years; controls: 24.5±3.9 years	LEO, radiographic assessment	CVD, (hsCRP)	Blood samples are often collected to determine additional risk factors for CVD.
De Oliviera et al. [20]	Brazil	CSS	2017	n=1346; 438 M/908 F	Only range between ≤18 and ≥60 years	AP, radiographic assessment	Prevalence of CAD	-
Virtanen et al. [21]	Finland	CSS	2017	n=120; 57 M/63 F	Cases: 53.0±2.7 years; controls: 51.4±2.9 years	AP, radiographic assessment	Prevalence of CVD	The majority of CVD cases were categorized as hypertensive (No particular number is given).
An et al. [22]	United States	CSS	2016	n=362; 98 M/266 F	Mean age: 49 years only	AP, radiographic assessment	Prevalence of HCA, CVD	Diabetes was another underlying illness in a portion of the participants.
Gomes et al. [23]	Brazil	Retrospective CS	2016	n=278; 143 M/135 F	55±16.8 years	AP, radiographic assessment	Incidence of CHD	Diabetes was another underlying illness in a portion of the participants.
Liljestrand et al. [24]	Finland	CSS	2016	n=508; 330 M/178 F	62.1±10.4 years	LEO, radiographic assessment	Prevalence CAD, ACS	Diabetes was another underlying illness in a portion of the sample; there was no clear breakdown of LEOs.
Lin et al. [25]	Taiwan	Retrospective CS	2015	n=283,590; 123,804 M/159,786 F	Only range between 20 and >60 years	incomplete RCT	1st assessment of CVD hospitalization	Diabetes was another underlying illness in a portion of the participants.
Costa et al. [26]	Brazil	CSS	2014	n=103; 52 M/51 F	Mean age: 61.9 years only	AP, radiographic assessment	Prevalence CAD	Diabetes was another underlying illness in a portion of the participants.
Petersen et al. [27]	Austria	CSS	2014	n=531	50±15.7 years	AP, radiographic assessment	The clinical, and radiographic volume of aortic atherosclerotic burden	Large number of Subgroups.
Willershausen et al. [28]	Germany	CSS	2014	n=497	51-83 years; Mean age: 62.3 years only	Prevalence of LEO	AMI	Periodontal lesions were also documented.
Pasqualini et al. [29]	Italy	Case-control	2012	n=100	Cases: 48±5.7 years; controls: 47±7.1 years	LEO, clinical, and radiographic assessment	AMI/UA	CD14 polymorphisms have been identified.

Primary Outcomes

In this review, only 14 articles have discussed the association between endodontic infections and CVD. The CVD entities and cases defined as outcomes in this review mostly included hard measures of CVD incidence/prevalence, overall coronary heart disease, involving myocardial infarction, unstable angina, coronary artery atherosclerotic/atherosclerosis burden, and coronary artery disease. Other alternative indicators for CVD outcomes, e.g., hypertension, flow-mediated dilation, and C-reactive protein elevation, were mentioned in only a limited number of studies [17,19,21]. In all but one investigation, a radiographic assessment confirmed chronic endodontic infection [16]. The 12 studies reported the assessment of “endodontic pathology,” “LEO,” and “AP” for the described endodontic status. In two studies of the included studies, the endodontic situation was described in terms of endodontic therapy and incomplete root canal treatment [16,25].

Secondary Outcomes

In this review, only eight studies investigated the association between LEO and CVD [18,20,22,23,26-29]. In these eight studies, most of the subjects with chronic endodontic infection/apical periodontitis had a greater chance of being diagnosed with CVD. The outcomes of these eight studies did not reveal a significant association between LEO and CVD. Although the study design may influence the association, the design of these eight studies does not reveal any effect on the association between LEO and CVD.

Quality Assessment

**Figure 2:**
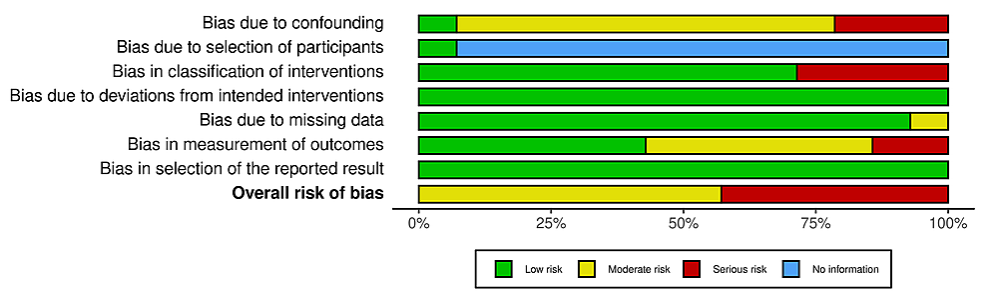
Overall risk of bias summary of all included studies

**Figure 3:**
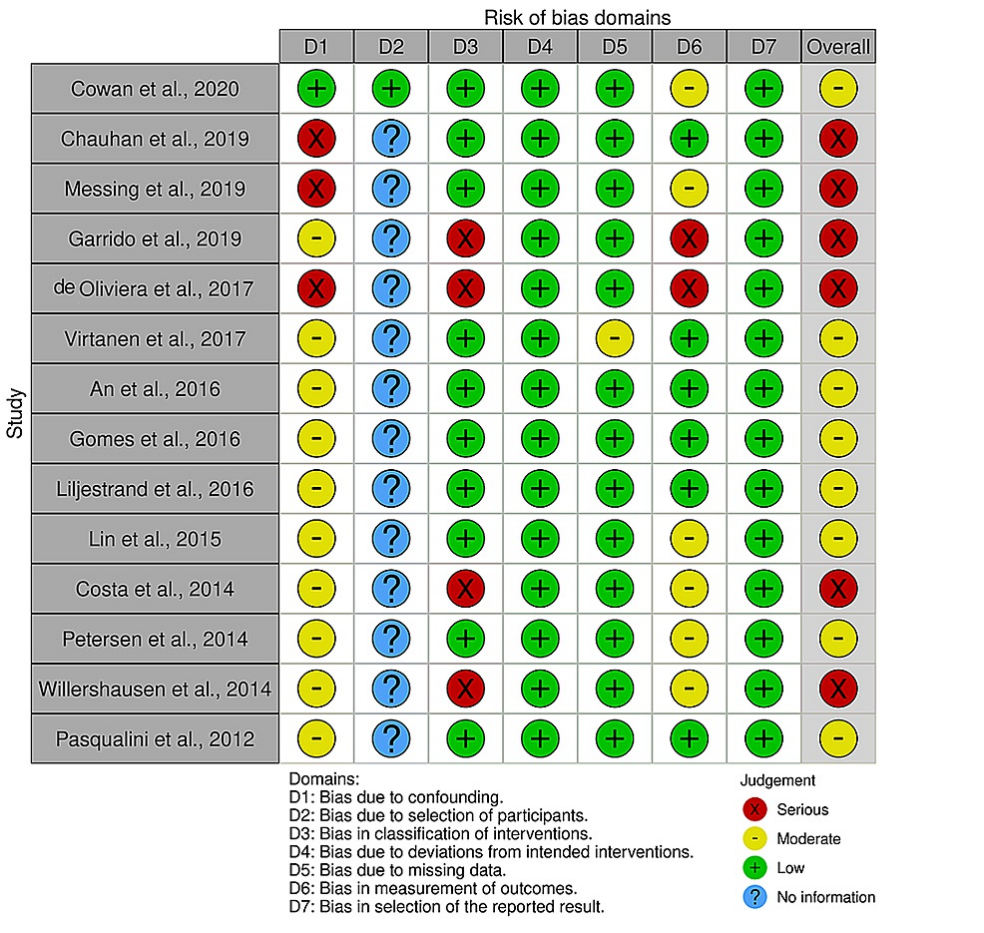
Risk of bias of the included studies (ROBINS-I tool) Source: Refs. [16-25]

The overall risk of bias was classified as serious (n=6) (42.86%) [17-20,26,28] and moderate (n=8) (57.14%) among the 14 articles analyzed (Figure 2) [16,21-25,27,29]. The overall risk of bias was not low in any of the included articles (Figure 2). The risk of bias for each study in all seven domains was rated as one of the four following categories: no information, low, moderate, and serious (Figure 3) [16-29].

Discussion

This systematic review aimed to investigate the association between chronic endodontic infections and CVD through observational studies published within the past 10 years (2011-2021) and meeting the inclusion criteria.

Endodontic infections and their influence on general health in the short- and long-term have been underway for the past 15 years, with growing intensity in the last five to six years. This finding could be supported by a recent report of a 10% increase in the estimated incidence of CVDs [30]; additionally, this finding is supported by WHO estimates of approximately 24 million individuals dying of CVD by 2030 [31]. Curiously, this disease has recently been implicated in the prognosis and pathogenesis of coronavirus disease 2019 (COVID-19), with an autopsy substances verifying a cytokine-mediated exacerbation and related inflammation in patients with CVD who had also tested positive for COVID-19, thereby highlighting the importance of further studies on factors potentially contributing to CVD, as seasonal epidemics or pandemics could conceivably place a heavier mortality burden [32].

Acute dental pulp inflammation caused by traumatic damage or deep caries can lead to pulp necrosis and infection in surrounding apical tissues, with an elevated potential to trigger the development of apical periodontitis proximal to contaminated internal tooth tissues [33]. Unsuccessful endodontic therapy can potentially predict the onset of apical periodontitis. Endodontic lesions develop as a result of microbial pathogen transfer from the contaminated pulp area to the periapical area of the tooth; hence, in essence, bone loss in the periapical area is also defined as the endpoint of an overactive immune response to inflammation [34]. Various inflammatory biomarkers are reportedly important in the pathophysiology and pathogenesis of endodontic origin lesions. The latest review described the foundation of the immune reaction to apical periodontitis as well as their biological processes [34].

Bacteria originating from infected tissues, in combination with the byproducts of systemic inflammation and the generation of granulomas, may be involved and could infiltrate systemic circulation and affect major arteries; thus, the possibility of a correlation with the occurrence of CVD could be assumed and considered biologically credible. Remarkably, attempts to decelerate the pathogenesis of apical periodontitis have a favorable impact on the biomarkers of endothelial dysfunction, as evidenced by effective endodontic therapy of afflicted teeth [35].

Current evidence for an association between CVDs and chronic endodontic infection/apical periodontitis has been weak. This finding was influenced by the design of the included studies as well as alternatives to reduce the quality of the evidence because of inter-study heterogeneity and publication bias issues. This suggests uncertainty regarding the impact estimate, and much required future investigations might very likely influence our understanding of this impact. Despite weak evidence of an association between CVD and chronic endodontic infection/apical periodontitis, clinical cardiologists must contemplate chronic endodontic infections as possible indicators for deleterious consequences in patients with CVD. Other oral pathological diseases, such as periodontal disease, have already been correlated in a comparable pattern [6]. Therefore, radiographic and clinical assessment of patients’ oral status, dentition, and different tissues are critical. Dental professionals and oral healthcare staff could be instrumental in preventing CVD on the basis of prognostic variables. In addition, radiographic examination of patients with CVD with standard panoramic radiographs can help efficiently identify the presence of periapical inflammatory lesions and counsel patients to perform a more thorough clinical investigation for evaluating whether these lesions are active or not, thereby reducing any future hazard for systemic side effects of inflammation and septicemia.

Strengths and Limitations

Our systematic review has some strengths; for instance, we searched four databases to avoid missing high-quality articles that would not appear or be indexed in specific databases. The included studies met strict inclusion criteria and were focused on only the past 10 years and were more updated than any other published article. Regarding the study limitations, most of the involved studies were of moderate to serious overall risk of bias. It was difficult to detect the publication bias in this review. Furthermore, we did not perform a meta-analysis because of heterogeneity concerns associated with patient characteristics and study settings, as well as confounding factors.

## Conclusions

Our findings indicate substantial uncertainty about evidence regarding an association between endodontic infections and CVD. Even though there appear to have been numerous efforts to clarify the causal process of these correlations, most investigation attempts have failed to demonstrate success, either as a result of study design and possible confounders or separately, but concurrently, acting factors persist to enforce a non-negligible degree of ambiguity on causation routes. Consequently, the detection of a higher risk for cardiovascular illnesses, as suggested by the current data synthesis, must be prioritized in the proper context, based on the identified limitations of the included original research methods. Further longitudinal clinical studies are required to determine the association between CVD and endodontic infections.
